# Data Twin-Driven Cyber-Physical Factory for Smart Manufacturing

**DOI:** 10.3390/s22082821

**Published:** 2022-04-07

**Authors:** Jung-Sing Jwo, Cheng-Hsiung Lee, Ching-Sheng Lin

**Affiliations:** 1 Master Program of Digital Innovation, Tunghai University, Taichung 40704, Taiwan; jwo@thu.edu.tw (J.-S.J.); hsiung@thu.edu.tw (C.-H.L.); 2 Department of Computer Science, Tunghai University, Taichung 40704, Taiwan

**Keywords:** Industry 4.0, Digital Twin, cyber-physical systems, digital manufacturing, data twin, cyber-physical factory, machine learning

## Abstract

Because of the complex production processes and technology-intensive operations that take place in the aerospace and defense industry, introducing Industry 4.0 into the manufacturing processes of aircraft composite materials is inevitable. Digital Twin and Cyber-Physical Systems in Industry 4.0 are key techniques to develop digital manufacturing. Since it is very difficult to create high-fidelity virtual models, the development of digital manufacturing for aircraft manufacturers is challenging. In this study, we provide a view from a data simulation perspective and adopt machine learning approaches to simplify the high-fidelity virtual models in Digital Twin. The novel concept is called Data Twin, and the deployable service to support the simulation is known as the Data Twin Service (DTS). Relying on the DTS, we also propose a microservice software architecture, Cyber-Physical Factory (CPF), to simulate the shop floor environment. Additionally, there are two war rooms in the CPF that can be used to establish a collaborative platform: one is the Physical War Room, used to integrate real data, and the other is the Cyber War Room for handling simulation data and the results of the CPF.

## 1. Introduction

Industry 4.0, the latest industrial revolution, has challenged the manufacturing industry with the advancement of digital technologies, and many governments have established their own strategic program to embrace the transformation and benefit from this wave [1]. However, even with its popularity in recent decades, at present, many enterprises are still in the early stages of the transition, and related technologies need to further develop before large-scale industry adoption [2].

Several emerging concepts, such as the Internet of Things (IoT) and Cyber Physical Systems (CPSs), have gained increasing attention across industries and are becoming more and more complex and relevant. The IoT is an infrastructure to link physical assets based on embedded sensors for the purpose of data transmission. It plays a key role in enhancing dynamic data and information processing capabilities, and affects operational agility [3]. The significance of its application area not only drives effective decision making, but also influences global economic systems and opportunities.

**Figure 1:**
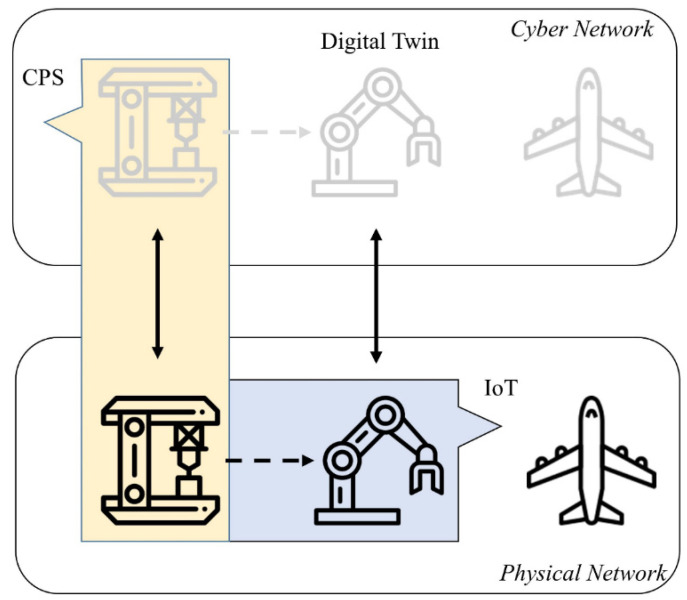
Two parallel networks and the relationship between IoT, Digital Twin and CPS.

The CPS has been proposed to bring the physical and the virtual world together for real-time data perception. According to the definition [4], “*cyber-physical systems are integrations of computation with physical processes. Embedded computers and networks monitor and control the physical processes, usually with feedback loops where physical processes affect computations and vice versa*”. With regard to the structure of CPS, as shown in Figure 1 (adapted from [5]), there are two parallel networks where a physical network interconnects field devices through IoT technologies, and a cyber network comprises a digital replica of the physical assets [6]. Specifically, the digital replica of a real-world “thing” can be characterized by Digital Twin.

The concept of Digital Twin was introduced to the general public by NASA as “*an integrated multiphysics, multiscale, probabilistic simulation of an as-built vehicle or system that uses the best available physical models, sensor updates, fleet history, etc., to mirror the life of its corresponding flying twin*” [7]. It is a model-based method to simulate the synchronization of the physical world and the related cyber world across the entire product lifecycle. Since then, researchers have started to investigate the integration of Digital Twin to realize CPSs for smart manufacturing.

Based on a physical product in the manufacturing environment, Digital Twin creates a highly accurate digital model to simulate the multiphysical behavior of the product in cyberspace, and performs advanced analytics to improve manufacturing processes by integrating historical and real-time data obtained from the product [8]. Note that the physical products are not merely limited to the physical assets such as machines or tools; they can be broadly extended to the manufacturing assets and manufacturing processes in high-value manufacturing industries, such as the automotive and aerospace sectors [9,10]. Despite the widespread acceptance of Digital Twin, and it being recognized as a huge movement for the manufacturing industry, there are still several challenges which require further study before it can be adopted into existing operational platforms. First, since the manufacturing industry often operates under constantly changing customer expectations and under strict environmental conditions, it is critical to build models that mirror this physical life in the digital world. More practical and economical models should be established to improve not only fidelity, but also flexibility and adaptability [11]. Second, although Digital Twin demonstrates the potential of increasing business impact by monitoring and controlling assets and processes, enterprises are required to consider the benefits of investment, as well as the risks and ongoing maintenance required. Complex systems will often be composed of complex twins and will therefore increase the complexity and cost of the Digital Twin. Since cost is always a critical consideration for business organizations, it is highly desired to have an efficient implementation with minimized investment without compromising the advantages of the Digital Twin [12]. This is especially important for small and medium enterprises (SMEs), due to their technical capabilities and limited resources.

With revolutionary advancements and substantial improvements in the development of neural network models, machine learning (ML) can be considered one of the most transformative technologies available, and has achieved considerable success in various domains—the manufacturing industry is no exception. Since ML is capable of discovering complex structures in high-dimensional data and providing useful abstraction for high-volume data, it is a good choice to address the aforementioned challenges. ML has been used to overcome human-in-the-loop digitalization challenges from a 3I (Intellect, Interaction and Interface) aspect [13]. In this paper, we go one step further and adopt an ML prediction model to represent physical products. By this computationally affordable and module-based development method, enterprises can be flexible with regard to starting the implementation according to their requirements, and can gradually transform towards smart manufacturing, no matter their size or type.

The contribution of this paper is two-fold:

- A lightweight and cost-effective solution: Building a Digital Twin is expensive and it is a cost burden for SMEs. Moreover, the industrial process is often complex, and some tasks are difficult to model, such as human behaviors and interactions. We introduce a lightweight service—Data Twin Service (DTS)—by applying ML to provide flexible modelling. Cyber-Physical Factory (CPF) is a form of microservice software architecture proposed to model the connection between the physical shop floor and the cyber space based on DTS;
- An application scenario in the aircraft industry: The aircraft industry requires small-volume-large-variety production, and the manufacturing process is complex and interactive. We present a representative scenario of CPF in the aircraft industry to demonstrate the orchestration of the proposed architecture and use this as an example to illustrate the method.

The remainder of this paper is structured as follows. Section 2 reviews key techniques relevant to the work of this paper. The proposed concept and software architecture are described in Section 3. A simulation case study and an application scenario are given to illustrate the use of the methodology in Section 4. In Section 5, we present conclusions and discuss future challenges and directions.

## 2. Related Work

In this section, we review some key technologies related to this research including Digital Twin, Cyber-Physical Systems and smart manufacturing.

The concept of Digital Twin was originally from a PLM lecture introduced by Dr. Michael Grieves in 2002, and was later formalized in one of his written papers as a “*virtual representation of what has been produced. Compare a Digital Twin to its engineering design to better understand what was produced versus what was designed, tightening the loop between design and execution*” [14]. Digital Twin has been applied in the manufacturing industry for various purposes. To support product lifecycles, Digital Twin is often developed for data monitoring to assist in supply chain management [15]. Another application of Digital Twin is for machine design. In order to obtain beneficial information for users without wasting resources, a Digital Twin can be created for a punching machine to simulate the interactive design of the NC machining process [16]. As human-centered design is one of the most promising approaches to improving the entire production process, a Digital Twin-based framework is often proposed to enhance the integration of ergonomics in the workplace to develop a new assembly line [17]. For example, for the improvement of production line performance, a Digital Twin of a cutting tool can be devised to incorporate the information based on the international standard (ISO 13399) to facilitate analysis and increase productivity [18]. The Digital Twin-based Process Knowledge Model (DT-PKM) incorporates geometric information, real-time process equipment status, and process knowledge big data to promote smart process planning [19]. Regarding the difficulties of Digital Twin, a viable challenge is to create a realistic high-fidelity model, especially for manufacturing products [20,21]. Since the Digital Twin can be supplied to enable intelligent decision-making, it is important to determine the interactions between components and existing systems. The selection of proper components and the introduction of new technology into complex systems have been investigated [22]. To enhance the operational flexibility, various enabling technologies and their impact on each other have been discussed [23]. Two case studies have been explored, where the first one combines IIoT and a collaborative robot, and the second combines IIoT and extended reality technologies. The Digital Thread is considered as a model to collect and share information through the different stages of supply chains and product lifecycles [24,25]. It also enables business management and full-process traceability in real-time collaborative development [26]. In additive manufacturing, the term Digital Thread is defined as “*the information and in-formation path that is gathered and stored when manufacturing a single part*” [27]. The combination of Digital Twin and Thread has been proposed in the shipyard industry to improve productivity and performance, where Digital Twin is used to represent the enterprise chains and Digital Thread is employed to integrate the enterprise data for digital continuity and accessibility [28].

The term Cyber-Physical Systems was introduced by Dr. Helen Gill in 2006 at the National Science Foundation (NSF) CPS Workshop. The concept integrates communication, computation and controllers in the system architecture of physical elements. The main objective is to add physical components to the computational network architecture for node communication, and one of the major applications of CPS has been in the manufacturing industry. The 5C architecture (Connection, Conversion, Cyber, Cognition and Configuration) is proposed to apply CPS in a manufacturing system and define a sequential workflow manner from the initial data acquisition to analytics and the final value creation [29]. To better implement the vision of CPS on the shop floor, three key technologies are discussed which include the interconnection among devices, industrial big data analysis for production process management, and intelligent decision-making. A three-layer architecture (physical, middleware and computation) is then presented and verified on a flexible automated production line [30]. Since blockchain technology has received significant focus in financial field, a conceptual three-level (Connection Net, Cyber Net and Management Net) blockchain framework is provided to resolve the inherent real-time implementation constraints of CPS in the application of manufacturing domains [31]. Bringing the human factor inside the cybernetic control loop presents a significant challenge to CPS, and the strengths and weaknesses of both robots and operators have been explored to compensate for the limitations of one another [32].

Based on the definition of the Smart Manufacturing Leadership Coalition (SMLC), smart manufacturing is *the dramatically intensified and pervasive application of networked information-based technologies throughout the manufacturing and supply chain enterprise* [33]. Since smart manufacturing is still an emerging trend and the core of the modern production environment, its effective realization is still a subject of research. Recent studies have focused on ML and AI methods because their advancements have already affected many research fields. According to the 5C architecture of CPS [29], an overview of the Industrial AI eco-system has been provided and a case study on the machine tool spindle of a CNC machine has been conducted [34]. The three main challenges (machine-to-machine interactions, data quality and cybersecurity) of adopting Industrial AI are discussed as well. To ensure SMEs are not left behind from the digital transformation, a Smart Manufacturing-related Information and Digital Technologies (SMIDT)-based approach has been used to integrate business operations [35]. The research also studied the determinants of SMIDT adoption and identified that a collection of technological, organizational, and environmental factors are key enablers. Several efforts have been made to integrate the Digital Twin into the smart manufacturing system design to achieve Industry 4.0 and promote the development of smart manufacturing [36,37].

## 3. Data Twin-Driven Cyber-Physical Factory

In order to solve the challenges discussed in Section 1, we present the concept of Data Twin to build the model for the physical products. Moreover, a microservice software architecture, Cyber-Physical Factory (CPF), is proposed to model the interaction between the physical world and virtual world based on Data Twin.

### 3.1. Data Twin

**Figure 2:**
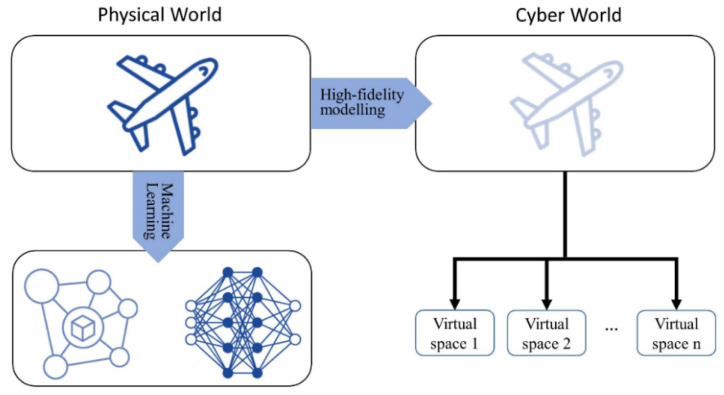
The comparison of Digital Twin (top-right) and Data Twin (bottom left).

**Figure 3:**
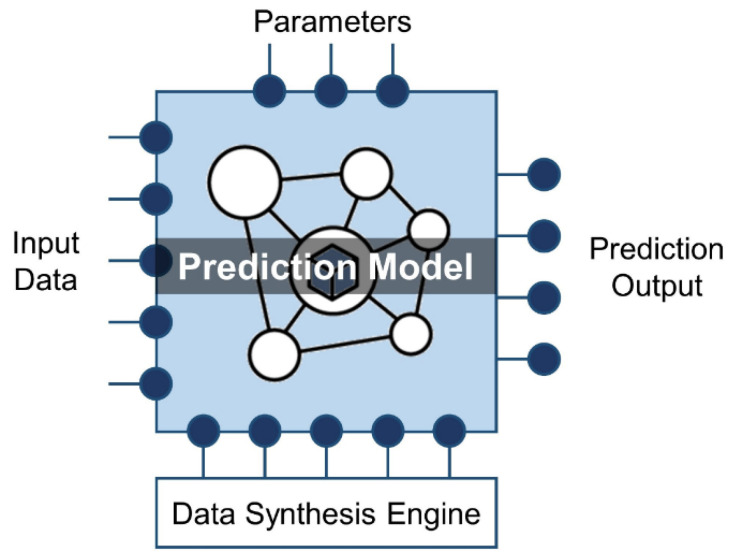
The architecture of Data Twin Service.

Digital Twin aims to create an accurate digital mapping of the physical product (Figure 2 top-right, adapted from [20]). However, it is difficult to implement for physical products in the complex manufacturing environment, especially with the human factor. The key challenges of Digital Twin in the manufacturing industry have been analyzed based on the academic literature and industrial knowledge base from five perspectives, including engineering, commercial, technology, data, and others [9]. There is still a gap to be bridged and a number of constraints to be considered before the actual realization. For most companies, massive amounts of data will be generated and stored for performing operational tasks. On the other hand, these data could be used to represent the task. Therefore, we propose a concept called Data Twin relying on data and ML to model real-world objects (Figure 2, bottom-left). The deployable service to support the simulation is known as the Data Twin Service (DTS), and consists of the following five components (Figure 3):

1. Input data: In order to accurately reflect the state, behavior or activity of the physical product, the Data Twin needs to be able to receive the operational data of the product. The data type could be streaming sensor data as well as the status of the product. There are three sources to obtain the data. The first one is acquired from the historical data, the second one is taken from real-time data, and the last one is derived from simulation data. A pre-processing approach could work in this component to clean the data and improve the understanding of the product;
2. Prediction model: The main purpose of Data Twin is to substitute the high fidelity in Digital Twin with the prediction model, so as to deal with automatic analysis and provide customized services. The prediction model could be implemented by the research fields of ML such as big data, data mining, pattern recognition and neural networks. It offers plug-in development and, most importantly, could consider the human factor in the loop for optimization and updating process flows [13]. In general, ML is classified into three major categories: supervised, unsupervised and reinforcement learning. Supervised learning optimizes a function that could map an input to an output based on the learning from training input/output pairs or the labeled data. Unsupervised learning aims at classifying the data into several clusters or uncovering hidden structures in unlabeled data. Reinforcement learning is a special ML technique where the agent learns to interact with the problem environments on the basis of maximizing long-term cumulative reward through the trial-and-error process. A suitable ML technique to be selected relies on the understanding of the data and problem domains;
3. Data Synthesis Engine: In ML, one of the main challenges is the severely limited training data, which is referred to as the cold start problem [38]. This is a practical concern in manufacturing enterprises when the level of digital competitiveness is low. Furthermore, we might only have scarce training data when implementing a new DTS. A Data Synthesis Engine is proposed to simulate data for the prediction model. Several manufacturing fields already apply simulation data to optimize the systems, such as the prediction of the remaining useful life [39], surface defect detection [40] and tool wear life modelling [41]. There are various open-source tools available for data synthesis, such as synthpop [42], simPop [43] and TGAN [44];
4. Parameters: There are two configurable parameter sets in the DTS setting. One is from the physical world products and the other is from the ML model. The physical behavior of factory equipment is affected by certain operating parameters. These parameters will not only determine the execution condition but also impact the performance of the involved system. In the ML area, hyperparameters are values or initial weights of the learning algorithm that are typically set by the practitioner based on domain knowledge and experience before the training process. These hyperparameters will then be used by the ML algorithm to fit the model parameters. Once these two parameter sets are determined, it is important for DTS to carry out the simulation test and iterate different configurations to optimize its performance and productivity;
5. Prediction output: The output format of the DTS can be analyzed from the user or system requirements. The prediction output could be simple values or as complex as some unstructured formats. Based on the input and output formats, the choice of prediction model can be determined to satisfy the constraints. The output of the previous DTS could be the input of the next DTS.

**Figure 4:**
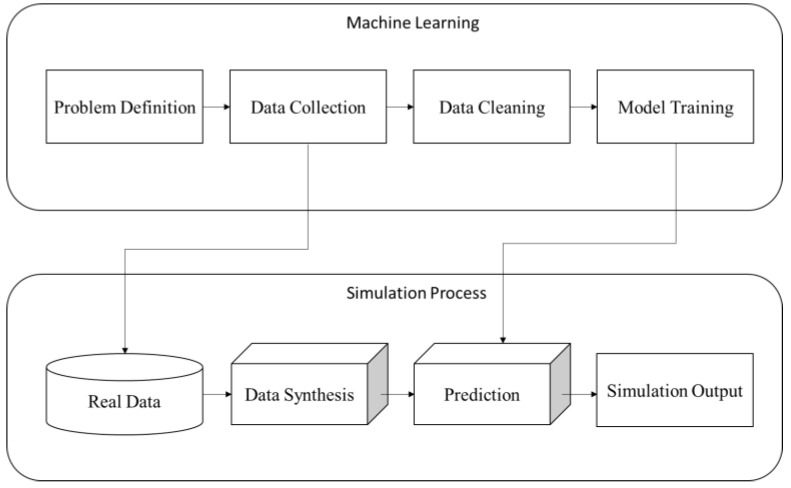
Workflow of Data Twin construction.

In Figure 4, we demonstrate the workflow of Data Twin construction containing machine learning and simulation processes. For a given operational task, we introduce ML techniques to model the problem. After ML is performed on real data and the trained model is obtained, we can use data synthesis to generate data and apply a trained model to make a prediction. It is worth noting that Data Twin could model not just physical products, but also human behaviors, which are difficult to construct by Digital Twin [45].

### 3.2. Cyber-Physical Factory

**Figure 5:**
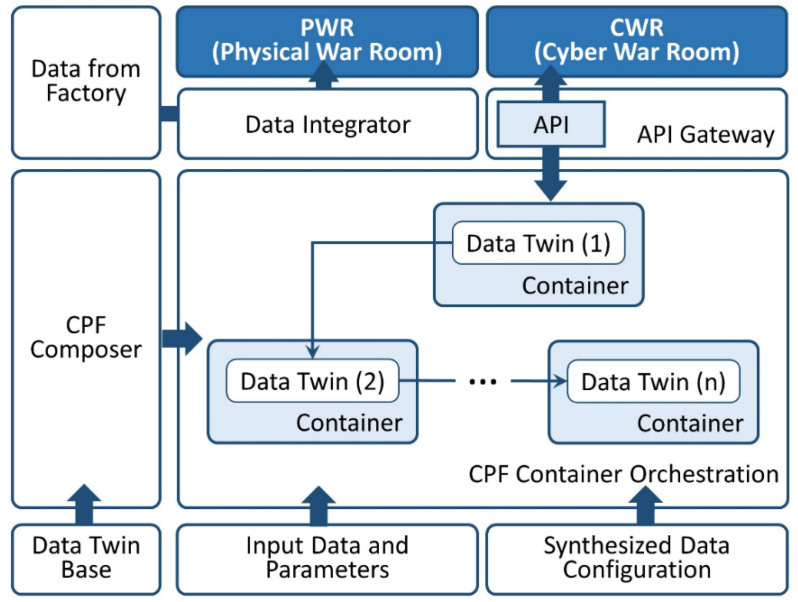
The architecture of the Cyber-Physical Factory.

This section mainly discusses applying the concept of Data Twin to build the architecture of a Cyber-Physical Factory (CPF). To develop and deploy the connection between the physical factory floor and the cyber computational space, the system architecture should satisfy two characteristics [29]: (1) the advanced connectivity to ensure the data delivery from the physical world and direct feedback from the cyber space; and (2) intelligent data management and analytics. Accordingly, our proposed architecture of the CPF provides an effective method and open communication, allowing the customization of suitable technology, the dynamic integration of various manufacturing resources, and frequent interaction operations. We detail the CPF software architecture, as well as its components, and the corresponding functions are as follows (Figure 5):

1. Data Twin Base: To conveniently manage the state of DTS, the Data Twin Base component is served as a repository for interfacing with DTS, which is uniquely identified by the ID. The supported operations include creation, query, update and deletion;
2. CPF Composer: This component is responsible for configuring the DTSs. Users are able to select desired DTSs and setup the corresponding five components of the DTS for the operational purpose. The assignment of data sources is described in the input data component of DTS (see Section 3.1) including real-time, historical, and synthesized data. The final and most important step is to establish the data and information flows between DTSs according to business demand. The integrated component will be loaded into the next CPF Orchestration module for the runtime execution;
3. CPF Orchestration: CPF Orchestration acts as the kernel of CPF where information and data are continuously being pushed to it from the physical units. In order to make each configured DTS offer high flexibility and high processing performance, container technology is adopted for the DTS residence with the advantages of convenient and consistent deployment. In addition to the management of context data, historical logs of DTS modifications and generated events will be gathered for the future analytics as well. The communication between services with other applications is through the API (Application Programming Interface) Gateway to simplify the message forwarding and authentication;
4. Cyber War Room (CWR): The key information which characterizes the product life cycle process could be visualized either by monitoring directly or predicting results based on ML. The CWR is used to demonstrate the digital replica of CPF. It can display information, alerts, indices, etc., to users based on the simulation results of each DTS in CPF. For example, in the fault detection scenario, if an alarm is triggered in CWR when the simulation value exceeds process operation limits, operators can investigate the cause of a fault, adjust the DTS setting to run the simulation, and perform the specific fix before the actual faults occur;
5. Physical War Room (PWR): The PWR reflects the real-time state of the shop floor in contrast to the CWR. For example, if the PWR shows a decrease in the production rate, operators can configure the DTS based on the observation to perform the optimization and then modify the related setup for the physical products to improve the production rate.

The CPF is implemented by the microservice architecture which describes a full application as a suite of services, where each DTS is running in its own container and communicating with others by lightweight mechanisms [46]. Compared with the traditional monolithic structure in which the application is packaged and deployed as a single executable with limited ability of self-recovery after a failure, each service within a microservice application, instead, can fail and be repaired independently. In general, the concept of microservice-based architecture originates from the classical Service-Oriented Architecture (SOA), but has been improved to allow better independence and deployment. The major benefits of adopting this architectural style in the digital factory are: (1) agility for a quick and economical development; (2) isolation and resilience for implementing independent service and self-recovery from the catastrophic event; and (3) elasticity with the ability to respond to workload fluctuations [47]. To make the whole process easier, a suitable environment should be provided such as a cloud platform that would support the CPF execution.

## 4. Simulation Case Study and Application Scenario

In this section, we demonstrate a case study of data synthesis for a Data Twin and a simplified application scenario in the field of aerospace manufacturing.

**Figure 6:**
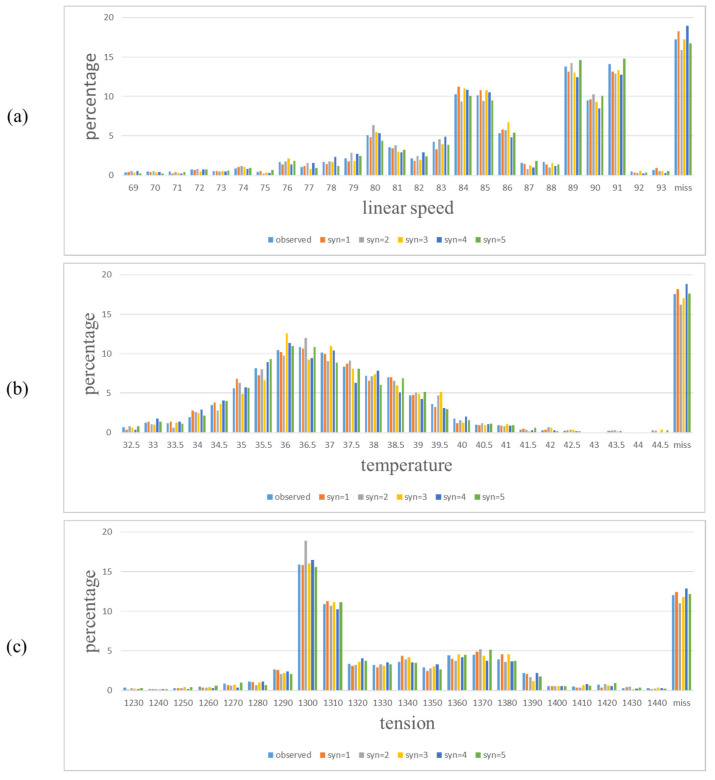
Three variables, linear speed (**a**), temperature (**b**) and tension (**c**) were selected for the distribution comparison between synthesis data and real one-month data.

In our industrial practice, one of the production lines dealt with textiles, and was required to detect defects for the purpose of condition monitoring. Based on the previous data, a Random Forest classifier with 81 variables to perform the detection already existed. To apply data synthesis for a Data Twin, we collected data for one month from the production line and used the existing Random Forest classifier to diagnose the defects. The predicted Matthews Correlation Coefficient (MCC) score was 0.2638 (denoted as MCC_b_). According to the one-month data, we then used synthpop [42] to generate 100 datasets. The existing Random Forest classifier evaluated each dataset and the MCC value was calculated. The statistics of the 100 MCCs are shown in Table 1. The first quartile is used to represent the middle value of the lower half of the data set, the second quartile is the median of the data set, and the third quartile is the middle value of the upper half of the data set. To determine whether the sample mean of 100 MCCs (denoted as MCC_a_) differed significantly from MCC_b_, one sample *t*-test was conducted at the significance level of 0.03. The null hypothesis assumed that there was statistically significant difference between MCC_a_ and MCC_b_, and the alternative hypothesis assumed that there was no significant difference between two values. We obtained the *p*-value of 0.00001704 and the null hypothesis was rejected, which implied that MCC_a_ was similar to MCC_b_. Three variables (linear speed, temperature and tension) were selected to compare the distributions between the simulation data and the one-month data. For each variable, we chose 5 datasets from 100 generated datasets (denoted as syn1 to syn5). From Figure 6, it can be observed that the synthesis data were similar to the one-month real data.

With the characteristics of high strength, stability and stiffness, the composite materials can be flexible for the airframe structure, and play a critical role in the aerospace and defense industry. To illustrate the proposed architecture, we introduced a simplified application scenario in the field of aerospace manufacturing. We used three representative stages of the bonding process to explain the use case, including: (1) lay-up, which is the process of depositing composite fibers onto a mold, layer by layer, in order to form a shape; (2) auto-clave-curing, which requires high temperature and pressure for the consolidation of polymer composites; and (3) assembly, which involves applying glues to connect the parts [48].

**Figure 7:**
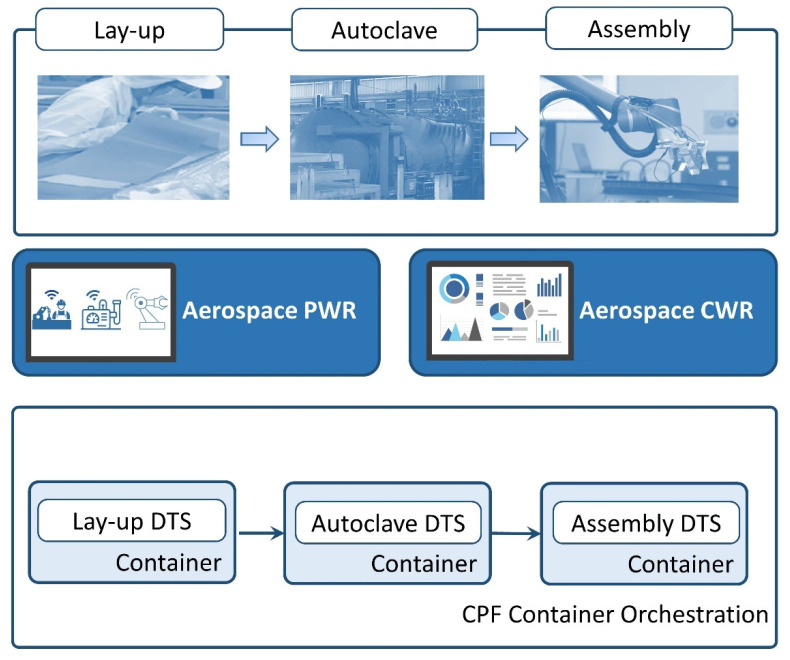
The three representative stages of the bonding process and the CPF in the aerospace manufacturing.

Three stages of the process on the shop floor are shown at the top of Figure 7, and the demonstration of the CPF is at the bottom of Figure 7, consisting of three connected DTSs, namely, lay-up, autoclave and assembly. Although manual lay-up is still the main approach to performing the task, exploring ways to obtain layup skills from experts is vital due to the increased market demand and lack of experienced operators. Hidden Markov models (HMMs) and three-dimensional Convolutional Neural Networks (CNN) are ML approaches used to recognize human activities and digitize behavior [45]. Modelling human-workpiece interactions constitutes the prediction model of the lay-up DTS, and includes monitoring the work of operators and transferring knowledge to novices. Predicting the curing degree difference in an autoclave to improve the uniformity is critical to optimally find the curing process parameter group [49,50]. Heating rate, holding time and holding temperature could be regarded as input parameters to estimate the maximum difference in the curing degree by ML and deep learning models to form the autoclave DTS. Applying glue to connect parts was previously dependent on the skills of the operators. Currently, we can obtain a better quality and reduce human operator’s workload through the use of a high-precision robot arm or cobot [51,52]. Moreover, to simultaneously ensure the gluing quality during the process, the visual inspection can be conducted by the vision-based deep learning approach to establish the assembly DTS [53]. Continuous updates of learning models in each DTS should be aligned with business requirements. The dashboard is a data and information visualization tool, allowing users to quickly understand the analytics that matter to their business objectives. The dashboard in PWR visualizes live data and statistics from an aerospace manufacturing shop floor, and presents related metrics and KPIs for the management at different scales and granularities. The dashboard in CWR was developed to publish the real-time results of each DTS, track the goal of each prediction model, and gain insight into performance. Additionally, the combination of these two dashboards establishes an agile and collaborative management platform to simulate business scenarios and expedite the modification of learning activities.

## 5. Conclusions

This paper presents a deployable service, Data Twin Service (DTS), based on machine learning, to model physical products and address the challenges of Digital Twin, which requires a high-fidelity twin for data acquisition. A microservice software architecture, the Cyber-Physical Factory (CPF), is proposed to simulate the shop floor environment based on the DTS. Aerospace manufacturing is taken as an example to illustrate the application of the CPF. Our approach proposes a view from a data angle to model industrial process and alleviate the complexity of Digital Twin for the manufacturing industry, including SMEs. The CPF architecture offers flexible implementation which is able to perform simulation and prediction. However, some challenges still need to be tackled and will be expanded in future research. First, more implementation and testing on different use cases are necessary to validate the robustness and effectiveness of the proposed framework. Second, the security issue in data exchange and storage should be considered. Data formats and communication protocols are important building blocks to solve this problem. Third, since the performance of our architecture heavily relies on the capability of machine learning models, investigating the advanced machine learning techniques of the DTS is crucial.

## Figures and Tables

**Table 1:** The statistics of 100 MCCs based on the synthesis data.

Min	1st Quartile	2nd Quartile	Mean	3rd Quartile	Max
0.1878	0.2406	0.2588	0.2603	0.2816	0.3245

## Data Availability

Not applicable.
